# Sensor Node for Remote Monitoring of Waterborne Disease-Causing Bacteria

**DOI:** 10.3390/s150510569

**Published:** 2015-05-05

**Authors:** Kyukwang Kim, Hyun Myung

**Affiliations:** 1 Department of Bio and Brain Engineering, Korea Advanced Institute of Science and Technology, 291 Daehak-ro, Daejeon 305-338, Korea; E-Mail: kyukwang.kim@gmail.com; 2 Department of Civil and Environmental Engineering, Korea Advanced Institute of Science and Technology, 291 Daehak-ro, Daejeon 305-338, Korea

**Keywords:** *E. coli*, coliform, chromogenic enzyme substrate assay, remote monitoring, water quality check

## Abstract

A sensor node for sampling water and checking for the presence of harmful bacteria such as *E. coli* in water sources was developed in this research. A chromogenic enzyme substrate assay method was used to easily detect coliform bacteria by monitoring the color change of the sampled water mixed with a reagent. Live webcam image streaming to the web browser of the end user with a Wi-Fi connected sensor node shows the water color changes in real time. The liquid can be manipulated on the web-based user interface, and also can be observed by webcam feeds. Image streaming and web console servers run on an embedded processor with an expansion board. The UART channel of the expansion board is connected to an external Arduino board and a motor driver to control self-priming water pumps to sample the water, mix the reagent, and remove the water sample after the test is completed. The sensor node can repeat water testing until the test reagent is depleted. The authors anticipate that the use of the sensor node developed in this research can decrease the cost and required labor for testing samples in a factory environment and checking the water quality of local water sources in developing countries.

## 1. Introduction

Coliforms are a group of oxidase-negative bacteria that produce acid from lactose or express β-galactosidase, and form yellow colonies of diverse shapes and sizes on membrane filters [1]. They can be found in the aquatic environments and in soil and vegetation as well as in the intestines of warm-blooded animals [2]. Detection of coliforms in water or food samples is important as they serve as a good indicator for measuring the presence of other fecal origin pathogenic bacteria such as *Salmonella* spp. or *Listeria* spp [3]. *Escherichia coli* (*E. coli*) is a representative species among coliform bacteria groups. Properties such as fast growing time, low biological hazards at high concentrations after culture (while certain strains of *E. coli* can be pathogenic [4] generally they are not), and well-studied physiological characteristics make *E. coli* a good marker bacteria for coliform detection.

Many molecular or immunological methods for detecting *E. coli* have been developed. Previous studies using Polymerase Chain Reaction (PCR), Enzyme-Linked Immunosorbant Assay (ELISA) [3], and antibody-conjugated gold nanoparticles (GNPs) [4] have been introduced. Each method has its respective strengths such as speed and accuracy; however, the process for detecting coliforms generally takes place in food factories, which have to deal with large numbers of daily samples, or in nations with developing economies where access to clean water is limited. Molecular methods are not suitable for these fields where minimum resource settings are required because the chemicals or enzymes used to run the process are expensive [2]. Paper-based point-of-care devices were suggested to reduce the burden in developing economies [5]. Paper-based fluidic devices are inexpensive and useful, but these devices are designed for single tests, and thus they are not appropriate for automated diagnosis processes that including multiple test runs.

**Figure 1:**
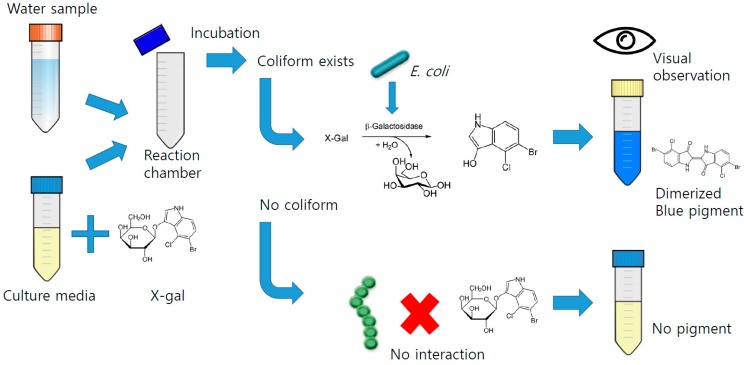
Process of chromogenic enzyme substrate assay.

The chromogenic enzyme substrate test using color indicating chemicals digested by the coliforms is one of the low cost methods used to detect *E. coli*. During culturing with chemicals called X-gal (also abbreviated as BCIG for 5-bromo-4-chloro-3-indolyl-β-D**-**galactopyranoside) or variants (Red-gal or Red colored X-gal for 6-chloro-3-indolyl-β-D-galactopyranoside, or MUG for 4-methylumbelliferyl β-D-galactopyranoside), coliforms digest X-gal variants and generate color pigments in a liquid broth, which can be visually detected by a color change of the media. Many commercial kits such as Colitag**^®^** (CPI International, Huntington Beach, CA, USA), Colilert**^®^** (IDEXX Laboratories, Westbrook, ME, USA), and Coliscan**^®^** (Micrology Laboratories, Goshen, IN, USA) are available for testing the presence/absence of coliform bacteria based on this principle, and are widely used and approved by diverse sanitation authorities, including the Republic of Korea Ministry of Environment [6] and the United States Environmental Protection Agency (US EPA) [2]. This method relies on bacterial growth in the edia and digestion of a given substrate, and thus the average time for the test is about 8 to 48 h. The time consumed for this test is relatively long compared to previously introduced methods. However, the chromogenic enzyme substrate method does not require a specific media (API-Analytical Profile Index 20 test [7] or MacConkey [8]) and it involves an easy procedure to prepare the test and a simple culture method that can be performed easily in the laboratory without expensive analysis devices. Intuitive classification of results also helps non-experts in bacterial diagnosis run this test easily. The overall process is described in Figure 1.

Although the chromogenic enzyme substrate test has many advantages, it is rather labor intensive as simple procedures have to be done repetitively. In a factory setting, the number of diagnosis samples may be large and handling daily portions is likely to be labor intensive. Water sources may be located far from residences in some countries with developing economies. In this case, the tester has to check each remotely located source daily. The assessment routines can be skipped for a few days, but decreasing the amount of labor in this way increase vulnerability to unexpected contamination by coliforms during the unchecked period.

In this study, we propose a low-cost sensor node system that can be placed in the water source or tanks in a factory and automatically and continuously samples water with a self-priming water pump, mixing with media containing a substrate, and checking the color change caused by bacterial growth. The tested water is purged after the test, and the new sample is drained from the source and the test is repeated until the loaded substrate media is depleted. An embedded Linux board and circuits are placed to access Wi-Fi to transmit data to the main server in the factory environment [9]. In the case of developing countries, individual users of different water sources interested in the presence of *E. coli* can place the sensor node in the source and check whether the source is contaminated.

## 2. Bacterial Culture and Chromogenic Enzyme Substrate Assay

Two *E. coli* strains were used for the experiment. *E. coli* K-12 MG 1655 strain was used as a positive control case; the presence of coliform bacteria in the water sample. *E. coli* DH-5 α strain was used to simulate the case of the existence of non-coliform bacteria (no β-galactosidase) as it has a Δ(*lacZ*) M15 mutation, and cannot digest X-gal and variants [10]. Even when coliform is not present, resident bacterial flora may be present in the water and grow in the testing device. *E. coli* DH-5 α strain was used to check whether false-positive cases can be filtered by a chromogenic enzyme assay. Bacterial strains were kindly provided by the Biomolecular Engineering Laboratory at the Dept. of Biological Science, Korea Advanced Institute of Science and Technology (KAIST), Korea.

X-gal (Takara, Otsu, Japan) was prepared by dissolving it in dimethyl sulfoxide (DMSO, Sigma Aldrich, St. Louis, MO, USA) to make a 0.5 M stock solution. The dissolved X-gal was stored at 4 °C and diluted to 1 mM concentration when used for the test. Five mL of LB broth (Duchefa Biochemie, Haarlem, the Netherlands) with 10 μL of X-gal stock was used as the assay media. Colitag^®^ was used for the control. A single Colitag^®^ pack was dissolved in 100 mL of distilled water (DW) and used to compare the results with the substrate media developed in this research. 

**Figure 2:**
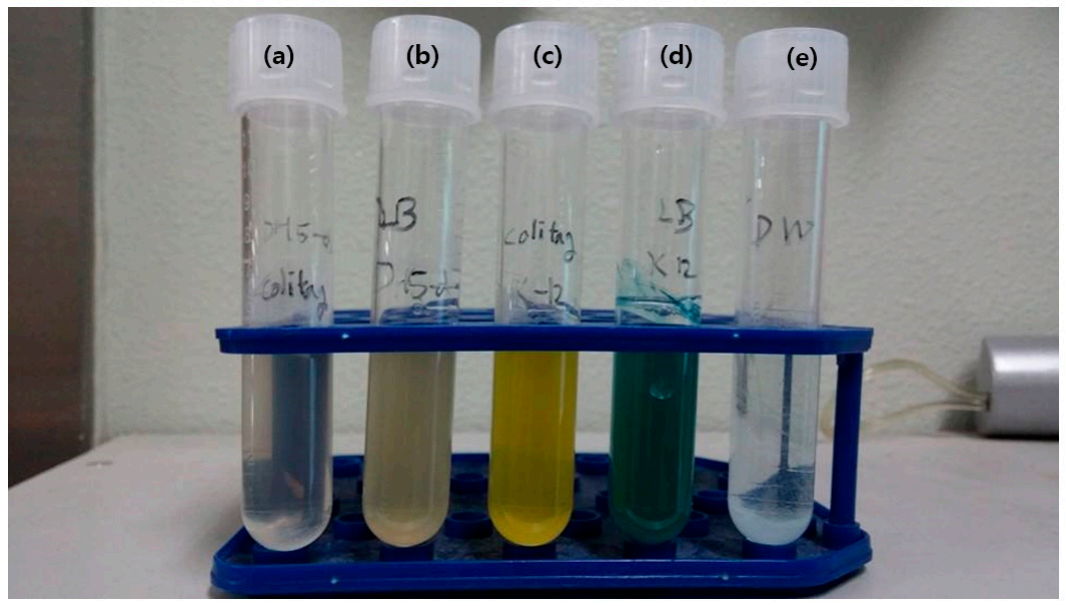
Chromogenic enzyme substrate assay. (**a**) *E. coli* DH-5 α with Colitag^®^; (**b**) *E. coli* DH-5 α with LB broth + X-gal; (**c**) *E. coli* K-12 with Colitag^®^; (**d**) *E. coli* K12 with LB broth + X-gal; (**e**) Negative control.

A total of five samples was prepared and incubated in a 36 °C incubator for 8 h. The results are shown in Figure 2. Figure 2a,b is *E. coli* DH-5 α strain with Colitag^®^ solution (a) and LB broth with X-gal; (b) Figure 2c,d is *E. coli* K-12 MG 1655 strain with Colitag^®^ solution; (c) and LB broth with X-gal; (d) Figure 2e is distilled water with X-gal as a negative control. The samples with *E. coli* DH-5α showed no specific color change after culturing compared to the samples incubated with *E. coli* K-12. Distinct yellow and blue pigments generation in Colitag^®^ and LB broth with X-gal can be observed when *E. coli* K-12 strains were included. The results show that the LB broth containing X-gal can distinguish coliform and non-coliform bacteria based on the presence of β-galactosidase activity. Visible and distinguishable color change makes it easy for non-experts in microbial diagnosis (for example, workers in factories or water quality testers in developing countries.) to check for the presence of coliform if an image of the culture bottle is provided to the user without requiring additional sensors or testing devices. Using less sensors for the detection period also increases the stability of the system as the calibration process required for sensor precision can be omitted, and the consumed power is also decreased. Furthermore, both the LB broth with X-gal and Colitag^®^ showed the same response against two *E. coli* strains, and testing reagents can be thus replaced by LB broth with X-gal to reduce the cost of consumable chemicals.

## 3. Circuit Design and Monitoring System Setup

**Figure 3:**
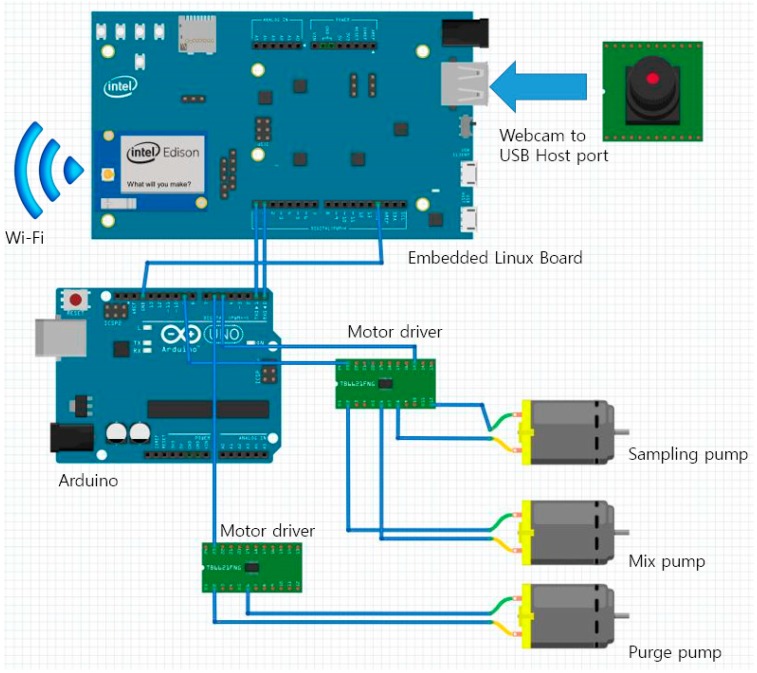
Circuit composition of the system. Power and ground lines are not shown for simplification of the diagram.

The sensor node was designed to operate three low voltage, self-priming DC water pumps (Seeed Technology Limited, Shenzhen, China) to perform an enzyme assay. The sampling pump drains water directly from the source to the reaction chamber. The reagent pump adds a specific amount of substrates with the culture media (Colitag^®^ or LB with X-gal), and the purge pump removes the tested water sample after the assay to prepare for the next step. The chromogenic process in the reaction chamber can be monitored by a camera attached to a sensor node, and streamed via a network connection to the end user. An Embedded Linux board (Intel® Edison development board from Intel, Santa Clara, CA, USA) with Arduino expansions were used as the main processor for network connections, camera streaming, and handling the user input/output (I/O) across Wi-Fi and a webpage. An additional Arduino board (Arduino, Ivrea, Italy) was attached for stable Pulse Width Modulation (PWM) control of the pump motors. The overall circuit diagram and connections are shown in Figure 3.

The Embedded Linux board and Arduino were connected via the UART port on the Arduino expansion board for motor control communication. Water pumps were connected to two dual channel motor drivers (TB6621FNG from Sparkfun, Niwot, CO, USA), controlled by Arduino. A webcam (Logitech, Lausanne, Switzerland) was directly connected to the Embedded Linux board via a USB host port in the Arduino expansion board. 5 V/3.3 V regulator board (AM-MP533 from NEWTC, Seoul, Korea) was used as a power source, connected to an external power supply or battery.

**Figure 4:**
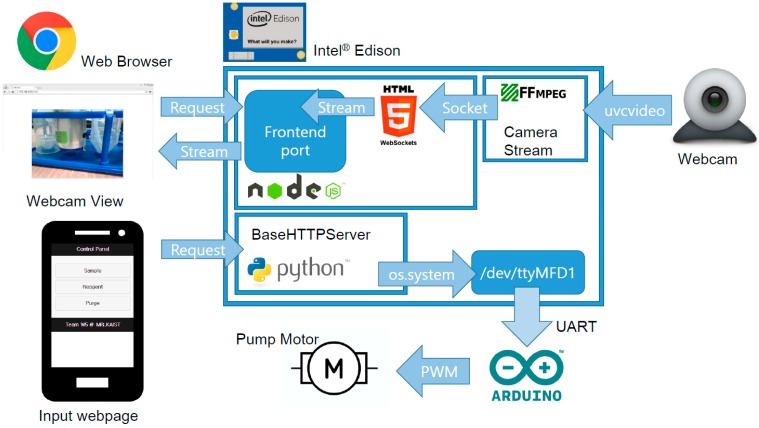
Overall architecture of the system.

A set of software packages was developed on the built sensor platform to monitor biological reactions and control water pumps through networks. The software architectures and data flow are shown in Figure 4, which was drawn by using Fritzing [11].

Streaming video from the USB webcam to the webpage was accomplished by adopting the “edi-cam” open source project [12]. The FFmpeg package and uvcvideo driver for Linux were used to obtain the video stream from the USB webcam. The obtained stream was sent to the canvas element of the webpage via a Websocket connection. A node.js powered frontend receives the user connection via the web browser, handles requests and Websocket streaming, and generates a live video stream at webpage. Google Chrome browser was selected in this research as it fully supports the most recent HTML5 and Websocket functions. Control panels also can be found at the webpage. Buttons and User Interface (UI) developed by jQuery Mobile are accessible through different ports from the video streaming page, powered by a multithreaded Python BaseHTTPServer module. While watching live webcam feeds via the video webpage, users can press the appropriate button to send a POST request to the Python server. Using the Python os module, commands transmitted with a POST request are sent to the UART channel driver. Intel® Edison has four UART drivers including channels for communication with an Arduino expansion board or a console connected by an OTG cable port. Driver ttyMFD1 is a UART channel that is mapped to the Rx and Tx ports of the Arduino expansion board. Appropriate settings for pin I/O mode, pullups, and multiplexer (MUX) of the device driver were set to open ttyMFD1 channel. Via pins connected to the external Arduino, data were transmitted to activate 78% duty Pulse Width Modulation (PWM) for 500 ms, which is appropriate for operating pump motors to move the required volume of water and the testing reagent to the reaction chamber.

## 4. Assembly of the Sensor Node and System Integration

**Figure 5:**
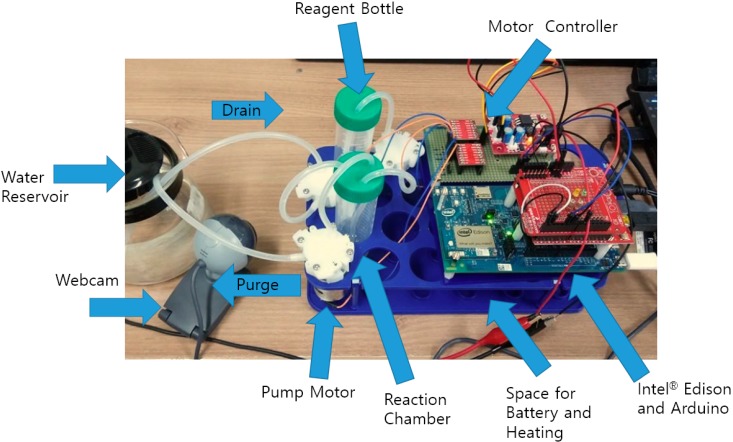
Assembled sensor node on the test bed.

The system components mentioned above were integrated to build a sensor node. Circuit boards and motors were attached to a plastic frame. 50 mL Falcon^®^ tubes were used for reagent storage and the reaction chamber. Silicon tubes with an outer diameter of 5 mm and an inner diameter of 3 mm were connected across the inlet and outlet of each liquid handling devices. A USB webcam was placed where the liquid flow of both the reagent bottle and the reaction chamber can be observed. A drain tube for sampling water into the reaction chamber and a purge tube that removes residuals of the reaction were placed in a water reservoir on the test bed to check liquid movement among the Falcon^®^ tubes. The assembled sensor node and the function of each device are illustrated in Figure 5.

**Figure 6:**
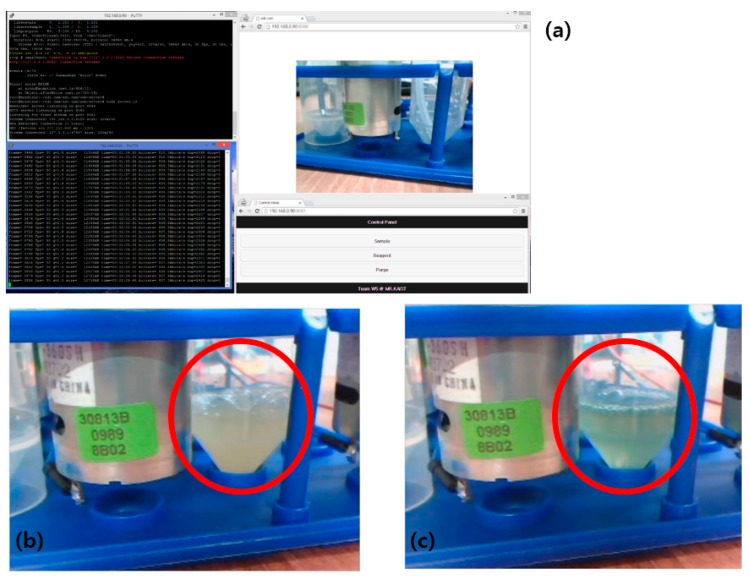
Operation of the sensor node. (**a**) Screenshot of operating sensor node. Console shows video stream and data request from user. Web pages show live image served to the end user and motor control buttons; (**b**) Streamed image of reaction chamber (red circled region) before reaction occurs; (**c**) Image of reaction chamber after reagent and *E. coli* incubated. Color in red circled region changes compared to Figure 6b.

The sensor node placed in the test bed was connected to a control PC with Wi-Fi connections. A wireless access point (AP) device was used to assign IP addresses to the sensor node and the PC. The end user manipulating the sensor node accesses the sensor node’s two servers with a browser via the given IP. Reception of commands and requests from the webpage is monitored via the console screen at the sensor node. The operation webpage of the sensor node is shown in Figure 6a.

After a function check, an experiment for detecting coliforms was carried out. DW with *E. coli* K-12 strain was injected to the reaction chamber, followed by injection of the testing reagent contained in the reagent bottle. Household hotwires were used as a thermal source (not shown in the figure for better visibility). Color change was monitored during overnight incubation.

Figure 6b shows an image of the reaction chamber bottle (red circled) before the overnight incubation process, which changed to the color shown in Figure 6c. The color of the solution in the reaction chamber changed as water containing *E. coli* was incubated together with the test reagent. As mentioned previously, the chromogenic enzyme substrate assay showed distinct color changes, and these changes could be easily detected on the live video stream on the webpage. The circled regions of Figure 6b,c shows a clear color change: before incubation, a RGB value of 169,166,149 was observed, and the RGB changed to 110,142,131 after incubation. G and B vectors did not show substantial changes (about 10~20 difference) but the R value dropped by more than 50. Calculating the R/G or R/B ratio can be helpful in scanning images from multiple sensor nodes automatically.

**Figure 7:**
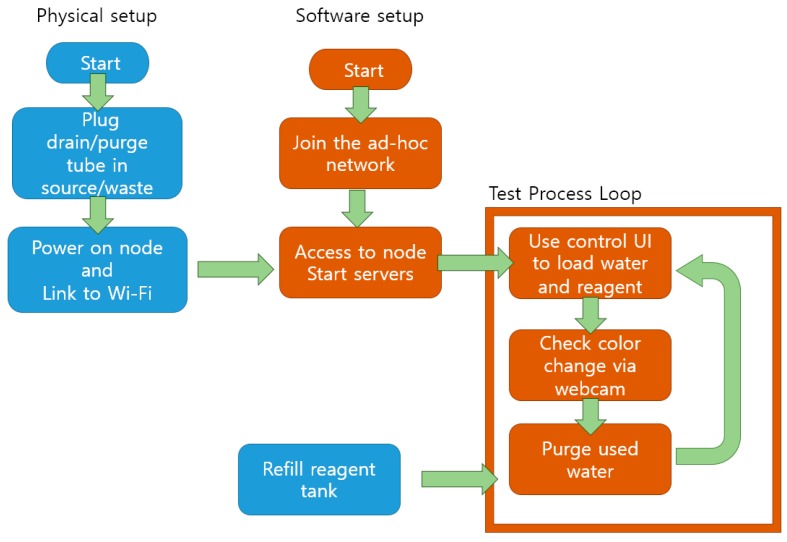
Operation scenario of the sensor node. Blue flowchart shows physical setup and processes while brown flowchart indicates software setup and operation.

Real-time video streaming also showed the current state of bottles without specific sensors. The amount of remaining reagent, the amount of liquid filled in the reaction chamber, and whether the reaction chamber is purged completely could be seen from the camera stream. The overall operation scenario is described in Figure 7. Consequently, fewer sensors are required for the system, thereby lowering the total cost for each sensor node (the prototype cost about 160 US dollars). The complexity and knowledge required for operation similarly are decreased.

## 5. Conclusions

In this research, a sensor node for remote monitoring of the presence of coliforms in water sources or food factories was developed. The chromogenic enzyme substrate assay method was used for the easy, low cost, and continuous detection of coliforms in samples. Water pumps controlled by Arduino and Intel^®^ Edison were equipped on each node; live video streaming and web-based motor control UIs are served by an internal server via a Wi-Fi network. Users can access each node placed at the remote sensing position easily via a webpage. Operation and monitoring are intuitive as live video images of the reaction chamber are provided. The current labor intensive chromogenic enzyme substrate assay process can be automated and simplified by using the system introduced in this study.

As a further development plan, we are considering issues such as wireless connectivity, a sustainable thermal control method, self-test functions, and easier UI. Many local communities in developing countries which have limited internet access use local *ad-hoc* networks with relay stations [13]. We used the embedded Linux board equipped with a Wi-Fi connection device to allow the node to join local wireless networks. Expanded connection functions such as a link to satellites are considered for local communities without these facilities.

The heating source and thermal control are important as they are directly linked to operation time and battery lifetime. The sensor node itself does not consume a lot of electricity; a household 5 V output DC adapter is enough to power the system, meaning that the heater consumes the largest portion of the total energy required. A solar power system to function without external power and continuous heating for the bacterial cultures is considered for the next development step. A simple box greenhouse is another good candidate for low-power thermal control.

Self-test functions are required to operate the system in the field for a long time. Feedback sensors such as devices for checking the liquid level of the reagent tank or testing the functionality of the pump motors are considered. Testing and increasing robustness of the software and circuits are important milestones, too.

The current system is not easy to use for users without knowledge about Linux systems. However it is simple enough to be operated with a little knowledge about Linux commands. Teaching students in developing countries about Linux with projects such as One Laptop Per Child (OLPC) project [14] or Raspberry Pi-based education [15] are now in progress. As the embedded Linux device used in this system is similar to the Raspberry Pi, we expect users who have completed these education courses can easily operate the sensor node. Automating the setup processes and development of an easier UI for simpler operation are now in progress.
